# Vaccinia Virus Protein C6 Is a Virulence Factor that Binds TBK-1 Adaptor Proteins and Inhibits Activation of IRF3 and IRF7

**DOI:** 10.1371/journal.ppat.1002247

**Published:** 2011-09-08

**Authors:** Leonie Unterholzner, Rebecca P. Sumner, Marcin Baran, Hongwei Ren, Daniel S. Mansur, Nollaig M. Bourke, Felix Randow, Geoffrey L. Smith, Andrew G. Bowie

**Affiliations:** 1 School of Biochemistry and Immunology, Trinity College Dublin, Dublin, Ireland; 2 Department of Virology, Faculty of Medicine, Imperial College London, St Mary's Campus, London, United Kingdom; 3 Medical Research Council Laboratory of Molecular Biology, Cambridge, United Kingdom; Saint Louis University, United States of America

## Abstract

Recognition of viruses by pattern recognition receptors (PRRs) causes interferon-β (IFN-β) induction, a key event in the anti-viral innate immune response, and also a target of viral immune evasion. Here the vaccinia virus (VACV) protein C6 is identified as an inhibitor of PRR-induced IFN-β expression by a functional screen of select VACV open reading frames expressed individually in mammalian cells. C6 is a member of a family of Bcl-2-like poxvirus proteins, many of which have been shown to inhibit innate immune signalling pathways. PRRs activate both NF-κB and IFN regulatory factors (IRFs) to activate the IFN-β promoter induction. Data presented here show that C6 inhibits IRF3 activation and translocation into the nucleus, but does not inhibit NF-κB activation. C6 inhibits IRF3 and IRF7 activation downstream of the kinases TANK binding kinase 1 (TBK1) and IκB kinase-ε (IKKε), which phosphorylate and activate these IRFs. However, C6 does not inhibit TBK1- and IKKε-independent IRF7 activation or the induction of promoters by constitutively active forms of IRF3 or IRF7, indicating that C6 acts at the level of the TBK1/IKKε complex. Consistent with this notion, C6 immunoprecipitated with the TBK1 complex scaffold proteins TANK, SINTBAD and NAP1. C6 is expressed early during infection and is present in both nucleus and cytoplasm. Mutant viruses in which the *C6L* gene is deleted, or mutated so that the C6 protein is not expressed, replicated normally in cell culture but were attenuated in two *in vivo* models of infection compared to wild type and revertant controls. Thus C6 contributes to VACV virulence and might do so via the inhibition of PRR-induced activation of IRF3 and IRF7.

## Author Summary

A key event in the innate immune response to virus infection is the detection of pathogen-associated molecular patterns (PAMPs) such as viral DNA and RNA by cellular pattern recognition receptors (PRRs). This leads to expression of interferon-β (IFN-β) by an infected cell. Many viruses have evolved mechanisms to evade the induction of IFN-β. Here a screen of poorly characterized vaccinia virus (VACV) proteins identified protein C6 as an inhibitor of IFN-β induction by PRRs. Data presented show that C6 prevents the activation of the transcription factors IRF3 and IRF7 by the kinases TBK1 and IKKε, which are key components at the point of convergence of several PRR signalling pathways. C6 interacts with the scaffold proteins NAP1, TANK and SINTBAD, which are components of the protein complexes containing TBK1 and IKKε, and this interaction might modulate the activity of these kinases. C6 is expressed early during infection and contributes to virulence because viruses that do not express C6 are attenuated in two *in vivo* models compared to wild type and revertant control viruses.

## Introduction

Mammalian cells respond to viral infection by producing pro-inflammatory cytokines and chemokines and also interferons (IFNs), of which type I IFNs, consisting of IFN-β and several IFNα proteins, are particularly important. IFN-α and IFN-β then act in an autocrine and paracrine manner to switch on hundreds of target genes which contribute to anti-viral innate immunity by blocking virus replication and alerting neighbouring cells to the danger of infection (reviewed in [1]). In addition to their role in innate immunity, type I IFNs also promote adaptive immune responses by priming T helper cells and cytotoxic T cells [2]. The initial production of type I IFNs is due to the activation of IFN regulatory factors (IRFs), and in particular IRF3, downstream of pattern recognition receptors (PRRs), which recognize viral DNA, RNA and proteins. PRRs that detect the presence of foreign RNA include the RIG-I-like receptors (RLRs) melanoma differentiation-associated gene 5 (MDA5) and retinoic acid induced gene I (RIG-I), which sense intracellular double-stranded (ds) RNA and single-stranded (ss) RNA containing a 5′ triphosphate, respectively [3]–[6]. Other PRRs that aid the detection of viruses include the endosomal toll-like receptors (TLRs), namely TLR3 which senses dsRNA, TLR7 and TLR8 which sense ssRNA and TLR9 which recognizes unmethylated DNA (reviewed in [7]). Intracellular DNA sensors such as AIM2, RNA polymerase III, DAI and IFI16 are also involved in sensing DNA viruses by recognizing the presence of dsDNA in the cytosol [8]–[15]. RNA polymerase III, DAI and IFI16 signal to cause type I IFN production, while AIM2 activates the inflammasome leading to processing of pro-interleukin (IL)-1β and release of IL-1β [9], [11], [12], [15]. RNA polymerase III is unusual in that it does not signal directly in response to DNA, but instead transcribes AT-rich DNA into RNA species, which are then recognized by RIG-I [8], [10].

The signalling pathways activated by the RLRs, the IFN-inducing intracellular DNA receptors and by TLR3 converge at the level of the kinases TNF receptor associated factor (TRAF) family member NF-κB activator (TANK)-binding kinase 1 (TBK1) and IκB kinase-ε (IKKε). These kinases exist in complexes with the scaffold proteins TANK, NAP1 (NAK-associated protein 1) or SINTBAD (similar to NAP1 TBK1 adaptor) [16]–[18]. To activate these kinases, RLRs and consequently RNA polymerase III signal via the adaptor protein MAVS (mitochondrial antiviral signalling) [19]–[21], other intracellular DNA sensors employ STING (stimulator of IFN genes) [22], [23], while TLR3 uses TRIF (TIR-domain containing adaptor molecule inducing IFN-β) [24], [25]. PRRs also require TRAF3 for the activation of TBK1 and IKKε (reviewed in [26]). Once activated by PRR signalling, TBK1 and IKKε phosphorylate IRF3, causing its translocation to the nucleus and the transcriptional activation of promoters containing appropriate binding sites, such as the IFN-β and CCL5 promoters, and a subset of promoters containing IFN-stimulated response elements (ISREs) [27], [28]. A related transcription factor of the IRF family, IRF7, also plays an important role in anti-viral responses and can be activated in a similar manner to IRF3 during viral infection [29]. However, while IRF3 is expressed constitutively, IRF7 is present at low levels in most cells, but is induced by type I IFNs in a positive feedback loop. Thus, IRF7 is particularly important for the continued expression of IFN-β during viral infection, and also contributes to induction of IFN-β by co-operation with IRF3 [29], [30]. In addition, IRF7 is essential for the induction of IFN-α genes that are not induced by IRF3 [29]. In plasmacytoid dendritic cells, an alternative TBK1- and IKKε-independent signalling pathway resulting in the activation of IRF7 is employed by TLR7, TLR8 and TLR9. These endosomal TLRs signal through the adaptor protein MyD88 (myeloid differentiation factor 88), leading to activation of the kinase IKKα which then phosphorylates IRF7 [29], [31], [32]. This is unusual, because in other PRR signalling pathways IKKα and IKKβ are involved in the phosphorylation of the inhibitor of NF-κB (IκB), causing its degradation and the subsequent activation of NF-κB. NF-κB is another transcription factor activated by PRRs, and is critical for innate immunity. NF-κB and IRF3 (or IRF7) co-operate with the activating protein 1 (AP-1) transcription factor family to induce the transcription of the IFN-β promoter.

A functional type I IFN response provides a potent means of controlling virus infections [33] and consequently viruses have evolved numerous counter-measures to stop the production or action of IFNs or IFN-induced anti-viral proteins (for review see [34]). These strategies include blockage of antiviral PRR signalling pathways (reviewed in [34], [35]). Viruses with a large DNA genome, such as poxviruses, encode an extensive array of immunomodulatory proteins. Vaccinia virus (VACV), an orthopoxvirus used as a vaccine to eradicate smallpox, has many immune evasion mechanisms, and these include intracellular proteins that block PRR signalling, secreted factors that sequester IFNs and proinflammatory cytokines, and proteins that inhibit the effector actions of an IFN response (reviewed in [36]). However, the exact function of many of the approximately 200 virus proteins remains unclear. Here, a functional screen was used to identify VACV proteins that inhibit the induction of the type I IFN response after PRR activation. It is shown that protein C6, the product of the *C6L* gene, is an inhibitor of IFN-β promoter activation. The C6 protein is a member of a family of VACV proteins that includes B14, A52 and K7 [37], [38]. The crystal structures of B14, A52 and K7 were solved [39], [40] and showed that they, and also VACV proteins N1 [41], [42] and F1 [43], adopt a Bcl-2-like fold. Functional characterisation showed that only F1 and N1 inhibit apoptosis [42], and consistent with this these proteins have a surface groove for binding BH3 peptides from pro-apoptotic Bcl-2 proteins [42], [43]. In contrast, proteins B14, A52 and K7 lack this groove and inhibit innate immune signalling pathways instead [40], [44]. Interestingly, the protein N1 is both anti-apoptotic and inhibits NF-κB activation induced by IL-1 [40], [42], [45].

In this paper we demonstrate that C6 inhibits the activation of IRF3 and IRF7 downstream of the kinases TBK1 and IKKε, while C6 does not inhibit signalling pathways using IKKα for IRF7 activation. Inhibition of IRF3 and IRF7 by C6 may be mediated by its interaction with the scaffold proteins TANK, NAP1 and SINTBAD. Consistent with the ability of C6 to inhibit IFN-β expression, recombinant viruses that do not express C6 are attenuated *in vivo* compared to the wild type and revertant viruses. C6 represents the first viral protein shown to target the TBK1 scaffold proteins.

## Results

### A screen of VACV proteins identified C6 as an inhibitor of IFN-β and CCL5 induction

**Figure 1:**
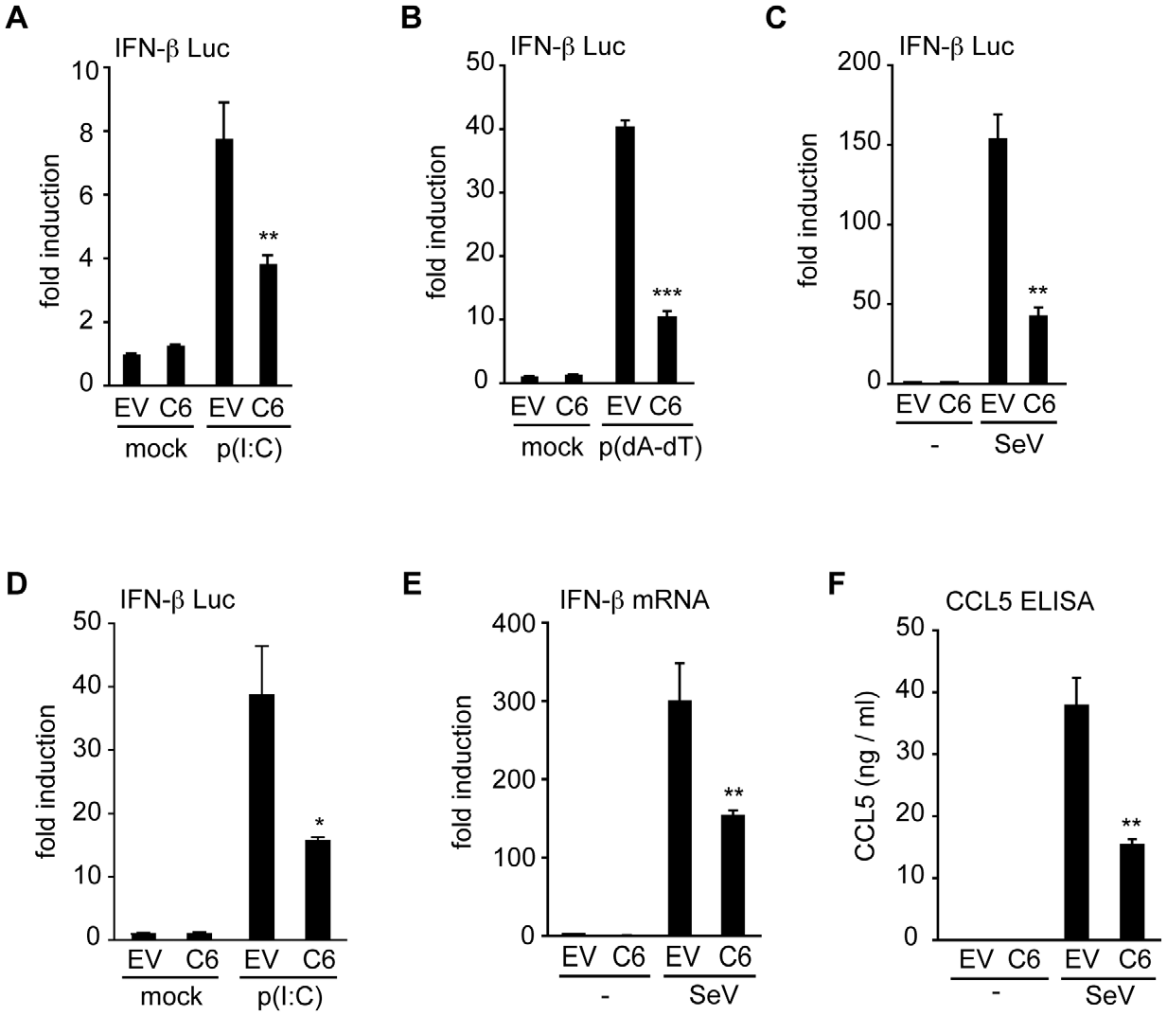
C6 inhibits IFN-β and CCL5 expression. (**A–C**) HEK293 cells were transfected with an IFN-β-promoter firefly luciferase reporter plasmid, a renilla luciferase transfection control, and a C6 expression plasmid (C6) or empty vector control (EV). Eight hours after transfection, cells were stimulated by transfection with 5 µg/ml poly(I∶C) (**A**), or 500 ng/ml poly(dA-dT) (**B**), with mock-transfected cells (mock) serving as control. In (**C**) cells were mock infected (-) or infected with Sendai virus. Firefly luciferase activity was measured 16 h after stimulation and normalized to renilla luciferase activity. (**D**) NIH3T3 cells were transfected with an IFN-β-promoter firefly luciferase reporter plasmid, a renilla luciferase transfection control and a C6 expression plasmid (C6) or empty vector control (EV). Cells were stimulated by transfection with 800 ng poly(I∶C) per well, and firefly and renilla luciferase activities were measured after 24 h. (**E, F**) HEK293 cells were transfected with empty vector (EV) or C6 expression plasmid (C6), and stimulated by infection with Sendai virus 24 h after transfection. After a further 24 h, IFN-β mRNA was measured by real-time PCR (**E**), or CCL5 protein secretion was measured by ELISA (**F**). Data are from one representative experiment of at least three, each performed in triplicate. Data are represented as mean ± SD. *p<0.05, ** p<0.01 or ***p<0.001 compared to EV.

To uncover novel VACV proteins that inhibit innate immune signalling pathways, a functional screen of proteins from VACV strain Western Reserve (WR) was used to identify those that inhibit type I IFN induction. For this, poorly characterized proteins encoded in the terminal regions of the VACV WR genome were selected, because these regions are rich in immunomodulatory proteins [46]. Proteins encoded within the highly conserved central region of the VACV genome were excluded from the screen, as were proteins with well-characterized functions, secreted proteins and those smaller than 8 kDa. This selection process identified 49 ORFs, and these were amplified from VACV WR strain genomic DNA, and cloned into mammalian expression vectors. Plasmids encoding these ORFs were transfected individually into HEK293 cells, and the effect on the IFN-β promoter following PRR stimulation was measured by reporter gene assays. ORF VACVWR022 (gene *C6L* in VACV Copenhagen strain) encoding the protein C6 emerged from this screen as an inhibitor of IFN-β promoter activation. Expression of C6 inhibited the activation of the IFN-β promoter by transfected poly(dA-dT) (which acts via intracellular DNA sensors) or poly(I∶C) (which acts via RLRs) in HEK293 cells (Figure 1A, B), and by infection of cells with Sendai virus which activates RIG-I signalling [4] (Figure 1C). C6 also inhibited poly(I∶C)-induced IFN-β promoter activation in mouse NIH3T3 cells (Figure 1D). Furthermore, the presence of C6 inhibited the expression of endogenous IFN-β mRNA in Sendai virus-infected cells (Figure 1E), as well as the secretion of the chemokine CCL5 from infected cells (Figure 1F). Thus, the C6 protein significantly reduced the expression of IFN-β and CCL5 after stimulation of PRRs by ligands or viral infection.

### C6 does not prevent the activation of NF-κB

**Figure 2:**
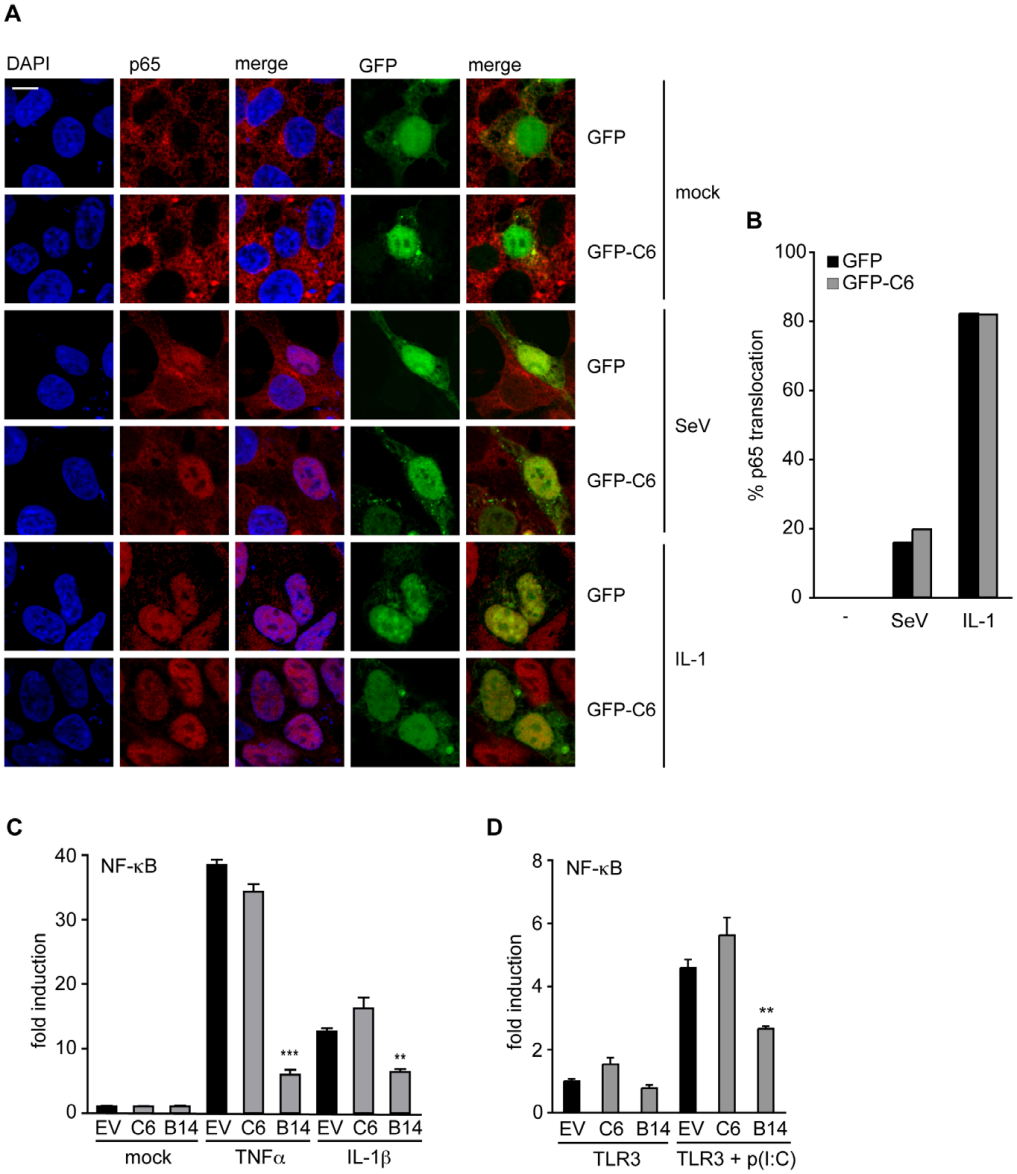
C6 does not inhibit the translocation or activation of NF-κB. (**A**) HEK293 cells were grown on coverslips and transfected with plasmids for the expression of GFP-tagged C6 or GFP as control. Twenty-four hours later, cells were mock treated (mock), infected with Sendai virus (SeV) for 6 h, or treated with 20 ng/ml IL-1β for 15 min. Cells were fixed and stained for endogenous NF-κB p65 (red). GFP or GFP-C6 are shown in green, and nuclear DNA is stained with DAPI (blue). (**B**) Cells expressing GFP or GFP-C6 from (**A**) were observed by confocal microscopy and scored for nuclear translocation of p65. At least 100 cells were counted for each sample. Data shown are from one representative experiment of at least three. (**C**) HEK293 cells were transfected with a firefly luciferase reporter plasmid under the control of an NF-κB-dependent promoter, a renilla luciferase transfection control, and a C6 or B14 expression plasmid or empty vector control (EV). Twenty-four hours after transfection cells were treated with 40 ng/ml TNF-α or 20 ng/ml IL-1β for 8 h. Firefly luciferase activity was normalized to renilla luciferase activity. (**D**) HEK293 cells were transfected with TLR3, an NF-κB-dependent firefly luciferase reporter plasmid, a renilla luciferase transfection control, and a C6 or B14 expression plasmid or empty vector control (EV). Eight hours after transfection cells were treated with 25 µg/ml poly(I∶C) for 16 h. Firefly luciferase activity was normalized to renilla luciferase activity. Data are represented as mean ± SD from one representative of at least three experiments each performed in triplicate. ** p<0.01 or ***p<0.001 compared to EV.

During viral infection, IRF3 and NF-κB co-operate to activate the IFN-β and the CCL5 promoters. IRF3 is phosphorylated by the kinases TBK1 and IKKε, leading to its dimerization and translocation to the nucleus. In a similar way, the kinases IKKα and IKKβ phosphorylate IκB, which then releases activated NF-κB and allows its nuclear accumulation. To investigate whether the signalling pathways leading to NF-κB or IRF3 activation are inhibited by C6, the translocation of the NF-κB subunit p65 from the cytoplasm to the nucleus was measured by confocal microscopy. For this, HEK293T cells were transfected with a plasmid expressing GFP-tagged C6, or a control plasmid expressing GFP for 16 h. The cells were then infected with Sendai virus for 6 h, or stimulated with IL-1 for 15 min, fixed and stained for endogenous p65. During Sendai virus infection, approximately 20% of control cells expressing GFP displayed p65 accumulation in the nucleus (Figure 2A, B), and the presence of GFP-tagged C6 did not affect the extent of p65 nuclear translocation. Furthermore, the expression of C6 did not affect the nuclear accumulation of p65 in cells stimulated with IL-1, an activator of NF-κB, which, regardless of whether the cells expressed GFP or GFP-tagged C6, caused p65 nuclear translocation in more than 80% of cells (Figure 2A, B).

The effect of C6 on the expression of a luciferase reporter gene under the control of an NF-κB-dependent promoter was examined next. Over-expression of C6 did not prevent activation of the NF-κB-dependent promoter stimulated by IL-1 or tumour necrosis factor (TNF)-α in HEK293 cells (Figure 2C), or by poly(I∶C) in HEK293 cells expressing TLR3 (Figure 2D). In contrast, the VACV protein B14, another member of the poxviral Bcl-2-like protein family, inhibited NF-κB promoter activation under these conditions (Figure 2C, D) as shown previously [47].

### C6 prevents the nuclear translocation of IRF3

**Figure 3:**
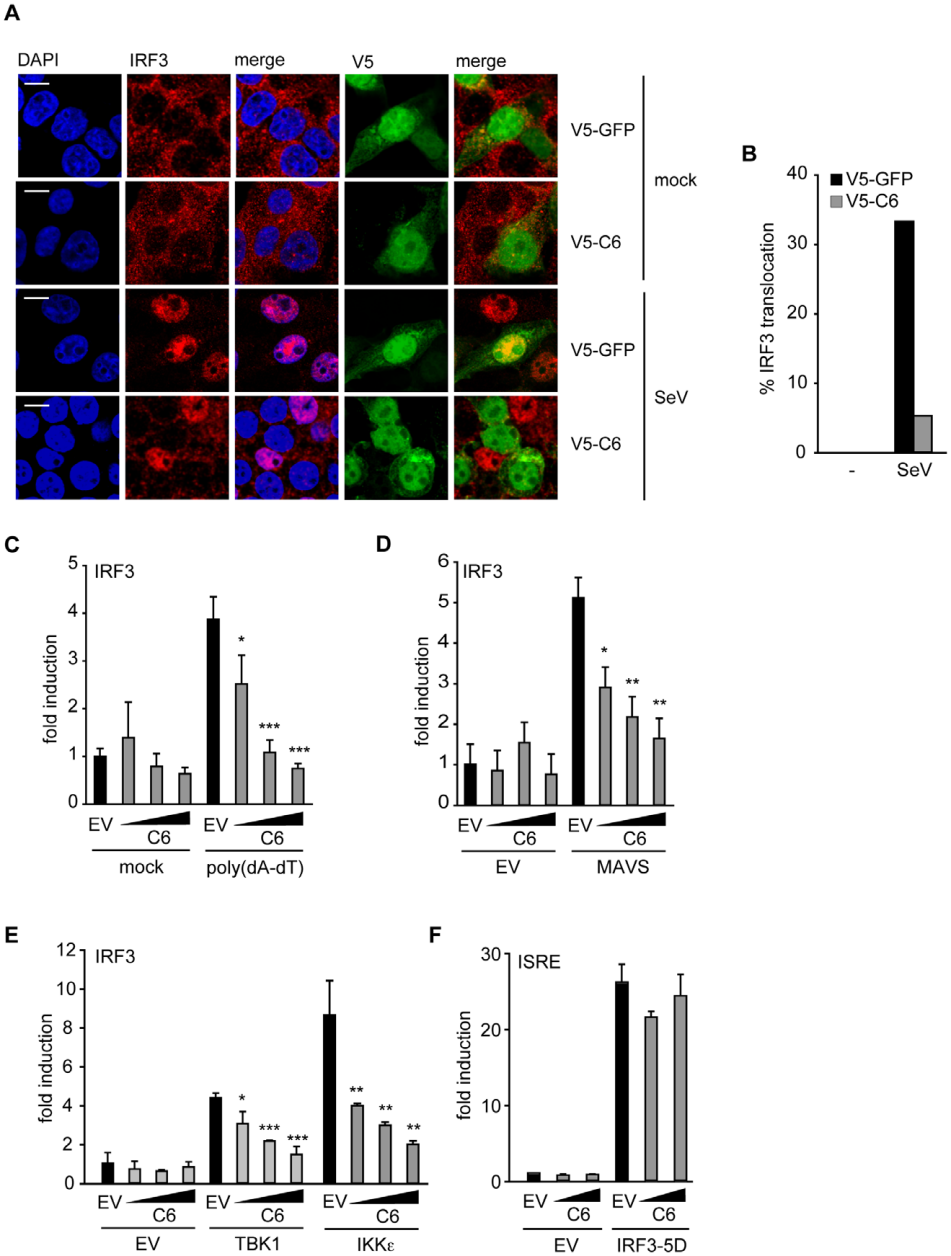
C6 inhibits the nuclear translocation and activation of IRF3. (**A**) HEK293 cells were grown on glass coverslips and transfected with plasmids for the expression of V5-tagged C6 or V5-tagged GFP as control. Twenty-four hours after transfection, cells were mock-infected (mock) or infected with Sendai virus (SeV) for 6 h. Cells were fixed and stained for endogenous IRF3 (red) and V5 (green). Nuclear DNA is stained with DAPI (blue). (**B**) Cells expressing V5–C6 or V5-GFP from (**A**) were observed by confocal microscopy and scored for nuclear translocation of IRF3. At least 100 cells were counted for each sample. Data shown are from one representative experiment of at least three. (**C–E**) HEK293 cells were seeded in 96-well plates, transfected with a pFR-firefly luciferase reporter plasmid under the control of the Gal4 promoter, a renilla luciferase transfection control, and an IRF3-Gal4 fusion plasmid. C6 expression vector (50, 100 or 150 ng, wedges) and empty vector (EV) were co-transfected per well. Expression plasmids for signalling factors (50 ng) were included in the transfection where indicated (**D and E**). In (**A**), cells were mock-transfected or transfected with 500 ng/ml poly(dA-dT) 8 h after the initial transfection. Cells were harvested 24 h after the first transfection, and firefly luciferase activity was measured and normalized to renilla luciferase activity. (**F**) HEK293 cells were transfected with a firefly luciferase reporter plasmid under the control of an ISRE element, a renilla luciferase transfection control, and a C6 expression construct or empty vector as above. The ISRE was driven by co-transfection of 2 ng of IRF3-5D. Cells were harvested 24 h after transfection, and firefly luciferase activity was measured and normalized to renilla luciferase activity. Data are represented as mean ± SD from one representative of at least three experiments each performed in triplicate. *p<0.05, ** p<0.01 or ***p<0.001 compared to EV.

The activation and nuclear translocation of IRF3 was investigated next. HEK293T cells transfected with V5-tagged C6 or V5-tagged GFP were infected with Sendai virus for 6 h, fixed and stained for IRF3. In cells infected with Sendai virus, IRF3 translocated to the nucleus in approximately 30% of cells expressing V5-tagged GFP (Figure 3A, B). However, in cells expressing V5-tagged C6, the translocation of IRF3 was impaired, and only 5% of C6-expressing cells displayed nuclear accumulation of IRF3 (Figure 3A, B). Similar results were obtained with GFP-tagged C6 (data not shown). This indicates that the inhibition of promoter induction by C6 is due to the prevention of the activation and/or nuclear translocation of IRF3, and not due to an effect on p65 activation.

### C6 inhibits IRF3 activation at the level of TBK1 and IKKε

To measure phosphorylation-dependent transactivation activity of IRF3, a luciferase-based IRF3 transactivation assay was utilized. This assay uses a fusion protein consisting of the DNA-binding domain of Gal4 and the transactivation domain of IRF3. When the IRF3 transactivation domain is phosphorylated by upstream signalling events, it induces expression of a luciferase reporter gene under the control of a Gal4-dependent promoter. Using this assay, C6 inhibited poly(dA-dT)-stimulated IRF3 transactivation (Figure 3C), providing further evidence that C6 inhibits the activation of IRF3 by PRRs. To determine which step of the signalling cascade that leads to IRF3 activation is targeted by C6, the ectopic expression of signalling proteins that act upstream of IRF3 activation was used to drive the IRF3 transactivation assay. The RLR adaptor MAVS and the kinases TBK1 and IKKε all promoted IRF3 activation in this assay when overexpressed (Figure 3D, E). Co-expression of C6 inhibited the activation of IRF3 in a dose-dependent manner in each case (Figure 3D, E), indicating that C6 acts at the level of these signalling components or further ‘downstream’ to prevent IRF3 activation.

To gain further mechanistic insight, the ability of C6 to inhibit the function of IRF3 once it is activated by phosphorylation was measured. To do this, a constitutively active form of IRF3 (IRF3-5D) was used in which serine to aspartate mutations mimic the phosphorylation of five key residues in the IRF3 sequence [48]. Over-expression of IRF3-5D induced the expression of a luciferase reporter driven by an ISRE element derived from the ISG15 promoter (Figure 3F), which was shown previously to be transcriptionally activated by IRFs [27]. C6 was unable to prevent the activation of the ISRE by over-expression of constitutively active IRF3 (Figure 3F), suggesting that C6 acts to prevent the activation of IRF3, but is unable to interfere with IRF3 function once it is activated by phosphorylation.

### C6 inhibits the activation of IRF7 by TBK1- and IKKε-dependent pathways

IRF7 is a transcription factor that is structurally and functionally related to IRF3 and also participates in the induction of the IFN-β promoter in response to PRR signalling (reviewed in [49]). IRF7 can be phosphorylated and activated by two distinct pathways. TLR3 and cytosolic PRRs, such as sensors of poly(dA-dT), act via TBK1 and IKKε, while the endosomal TLRs TLR8 and TLR9 activate IRF7 using a signalling pathway independent of TBK1 and IKKε, but involving MyD88 and IKKα [29], [32], [50].

**Figure 4:**
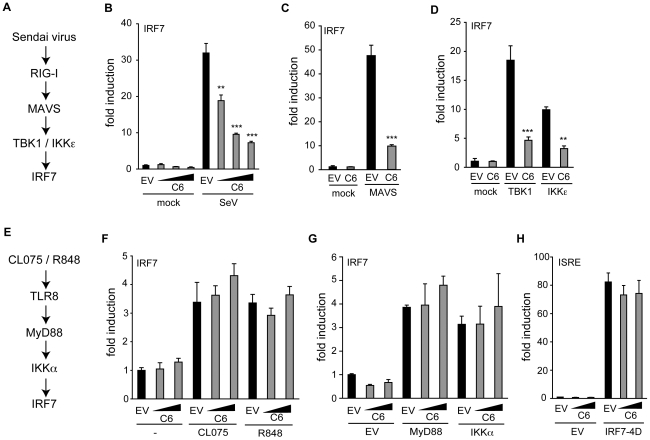
C6 prevents IRF7 transactivation stimulated by TBK1-and IKKε-dependent pathways. (**A,E**) Schematic outline of a TBK1/IKKε-dependent (**A**) and –independent (**E**) pathway resulting in the activation of IRF7. (**B–D, F, G**) HEK293 cells were transfected with pFR-firefly luciferase under the control of the Gal4 promoter, a renilla luciferase transfection control, and an IRF7-Gal4 fusion plasmid. C6 expression vector and empty vector (EV) were co-transfected as indicated. In (**B**), cells were infected with Sendai virus (SeV) for 16 h. (**C, D, G**) Expression plasmids (50 ng) for signalling factors were included in the initial transfection as indicated. (**F**) HEK293 cells expressing TLR8 were stimulated with 2.5 µg/ml CL075 or 1 µM R848 for 16 h. (**H**) HEK293 cells were transfected with a firefly luciferase reporter plasmid under the control of an ISRE element, a renilla luciferase transfection control, and a C6 expression plasmid or empty vector. The ISRE was driven by co-transfection of 2 ng of IRF7-4D. In all cases, cells were harvested 24 h after the first transfection, Firefly luciferase activity was measured and normalized to renilla luciferase activity. Data are represented as mean ± SD from one representative of three experiments, each performed in triplicate. ** p<0.01 or ***p<0.001 compared to EV.

The ability of C6 to inhibit the TBK1/IKKε-dependent and -independent signalling pathways to IRF activation was compared by employing a luciferase-based IRF7 transactivation assay. Activation of the TBK1/IKKε-dependent pathway following infection of HEK293 cells with Sendai virus (outlined in Figure 4A) was inhibited by C6 (Figure 4B), as was the activation of IRF7 by the downstream signalling components MAVS, TBK1 and IKKε (Figure 4C, D). To determine whether C6 could inhibit the TBK1/IKKε-independent pathway to IRF7 activation, HEK293 cells stably expressing TLR8 were used. Stimulation of TLR8 by the agonists CL075 or R848 activates IRF7 via MyD88 and IKKα (outlined in Figure 4E), however, this was not inhibited by C6 (Fig. 4F). Similarly, C6 was unable to inhibit IRF7 activation following over-expression of MyD88 or IKKα (Figure 4G). As observed for IRF3, C6 was unable to inhibit the activation of the ISRE element in response to constitutively active IRF7 (IRF7-4D, Figure 4H).

Taken together, these data indicate that C6 inhibits the activation of IRF3 and IRF7 by TBK1- and IKKε-dependent signalling pathways, implying that C6 acts on these kinase complexes, rather than acting on the transcription factors directly.

### C6 function is conserved in monkeypox virus

Published sequence data show that C6 has orthologues in other orthopoxviruses (OPVs), the capripoxvirus and deerpoxvirus (www.poxvirus.org), and within the OPV genus the conservation is high (89–97% amino acid identity). To investigate if C6 function is also conserved, the ability of the C6 orthologue from monkeypox virus (MPXV) strain ZAI 1979-005 (92% amino acid identity) to inhibit poly(dA-dT)-induced IRF3 transactivation was investigated (Figure S1A). The MPXV ORF encoding the C6 orthologue was amplified from DNA extracted from MPXV-infected HeLa cells. When a MPXV C6 expression vector was transfected into cells, the MPXV C6 protein, like the VACV C6 protein, inhibited the pathway at the level of TBK1 and IKKε, because MPXV C6 inhibited the activation of IRF3 caused by the over-expression of either of the two kinases, or of the adaptor protein MAVS (Figure S1B). Thus, the MPXV C6 orthologue behaved like VACV C6 in the assays tested.

### C6 associates with the TBK1 scaffold proteins SINTBAD, NAP1 and TANK

**Figure 5:**
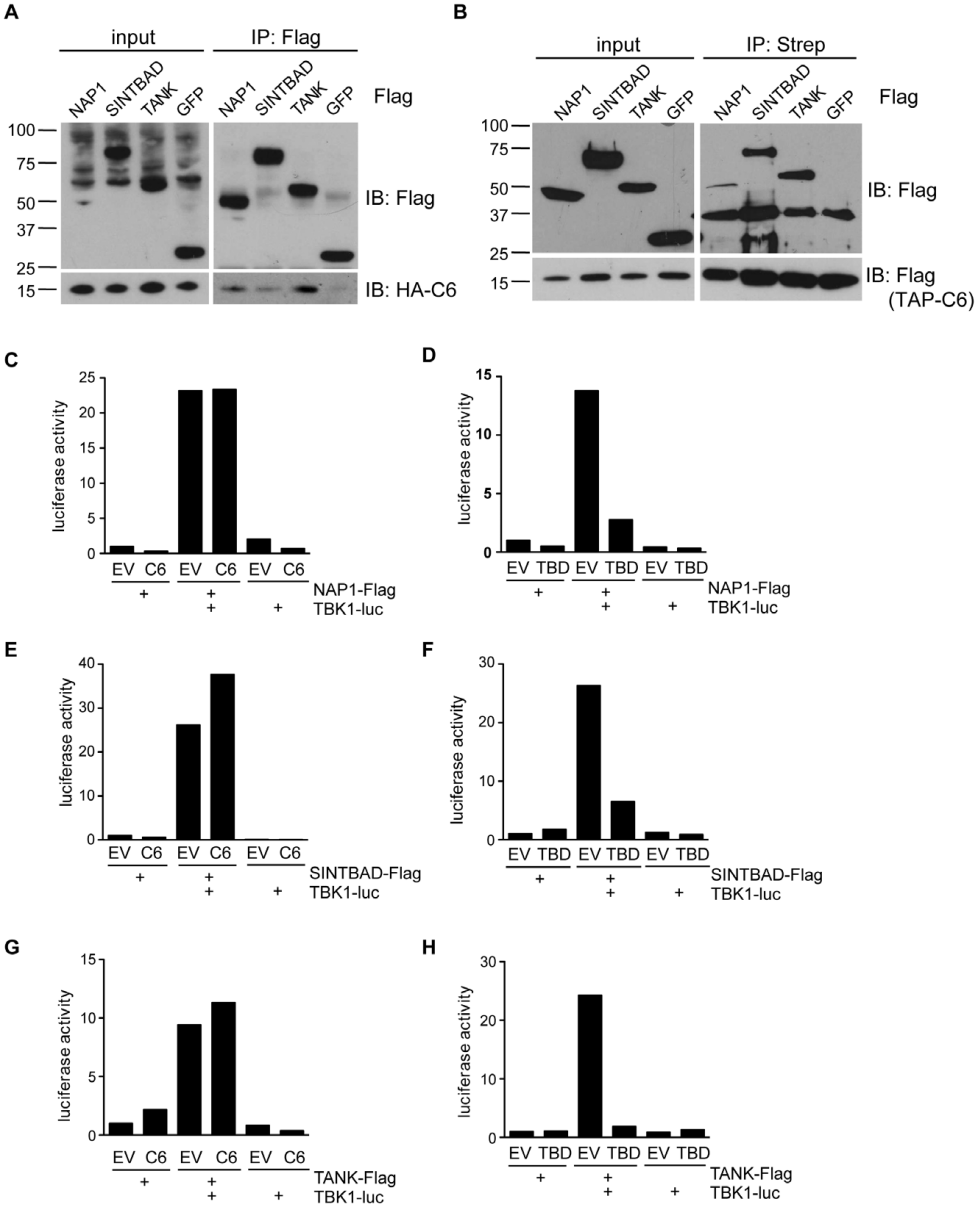
C6 interacts with NAP1, TANK and SINTBAD. (**A**) HEK293 cells were grown in 10-cm dishes and transfected with 10 µg plasmids encoding FLAG-tagged NAP1, SINTBAD, TANK or GFP as indicated. After 48 h, cells were infected with recombinant VACV expressing HA-tagged C6 (2 p.f.u. per cell) for 16 h. Cell lysates (input) were subjected to immunoprecipitation (IP) with anti-FLAG M2 agarose beads. Proteins were separated by SDS-PAGE and detected by immunoblotting as indicated on the right. (**B**) HEK293 cells were grown in 10-cm dishes and transfected with 5 µg plasmids encoding FLAG-tagged NAP1, SINTBAD, TANK or GFP and 5 µg TAP-tagged (consisting of FLAG and Strep epitopes) C6 as indicated. After 48 h cell lysates (input) were subjected to immunoprecipitation (IP) with Streptavidin agarose beads. Proteins were separated by SDS-PAGE and detected by immunoblotting as indicated on the right. (**C–H**) HEK293 cells were grown in 6-well plates and transfected with 0.5 µg luciferase-tagged TBK1 expression plasmid, 0.5 µg FLAG-tag expression plasmid and 3 µg C6 or TBD expression vector or empty vector (EV) as indicated. Cells were harvested after 24 h and subjected to immunoprecipitation with anti-FLAG antibody. Immunoprecipitated protein complexes were eluted with FLAG peptide and co-immunoprecipitated luciferase activity was measured. Data are representative of at least three experiments.

To investigate how C6 antagonises activation of the pathway at the level of the TBK1- and IKKε-containing complexes, interactions between C6 and components of the kinase-containing complexes were sought by immunoprecipitation. HEK293 cells were transfected with plasmids encoding FLAG-tagged proteins and then infected with a VACV expressing HA-tagged C6. Immunoprecipitation with anti-FLAG antibody co-precipitated C6 with the scaffold proteins NAP1, SINTBAD and TANK but not with a FLAG-tagged control protein, FLAG-GFP (Figure 5A). In contrast, no interaction with TBK1 or IKKε was detected (data not shown). A reciprocal immunoprecipitation using lysates from cells over-expressing Streptavidin-tagged C6 and FLAG-tagged scaffold proteins also showed an interaction between C6 and the adaptors since immunoprecipitation of C6 co-precipitated NAP1, SINTBAD and TANK, but not GFP (Figure 5B).

It has been proposed that TBK1 and IKKε form distinct complexes, either as homodimers or heterodimers, which would contain a specific scaffold protein (namely TANK, NAP1 or SINTBAD [17], [51], [52]). To investigate whether the interaction between C6 and NAP1, TANK or SINTBAD affected the formation of signalling complexes, the scaffold-kinase interactions were investigated in the presence or absence of C6. A LUMIER interaction assay was used, in which a FLAG-tagged scaffold protein and luciferase-tagged TBK1 were co-expressed in the presence or absence of C6, and the amount of luciferase co-immunoprecipitated with the FLAG-tagged allele was quantified [17], [53]. C6 did not prevent the association between TBK1 and NAP1, SINTBAD or TANK (Figure 5C, E, G). In contrast, expression of isolated TBK1-binding domains (TBDs) inhibited the formation of the scaffold-kinase complexes (Figure 5D, F, H) as described previously [17]. Similar results were obtained for the interactions between the scaffold proteins and IKKε, which were not disrupted by C6 (Figure S2A–C). Thus, C6 appears to associate with the scaffold proteins TANK, NAP1 and SINTBAD, without disrupting the formation of the signalling complexes containing the kinases TBK1 or IKKε.

### C6 is expressed early during infection by VACV and localises to both the cytoplasm and nucleus

**Figure 6:**
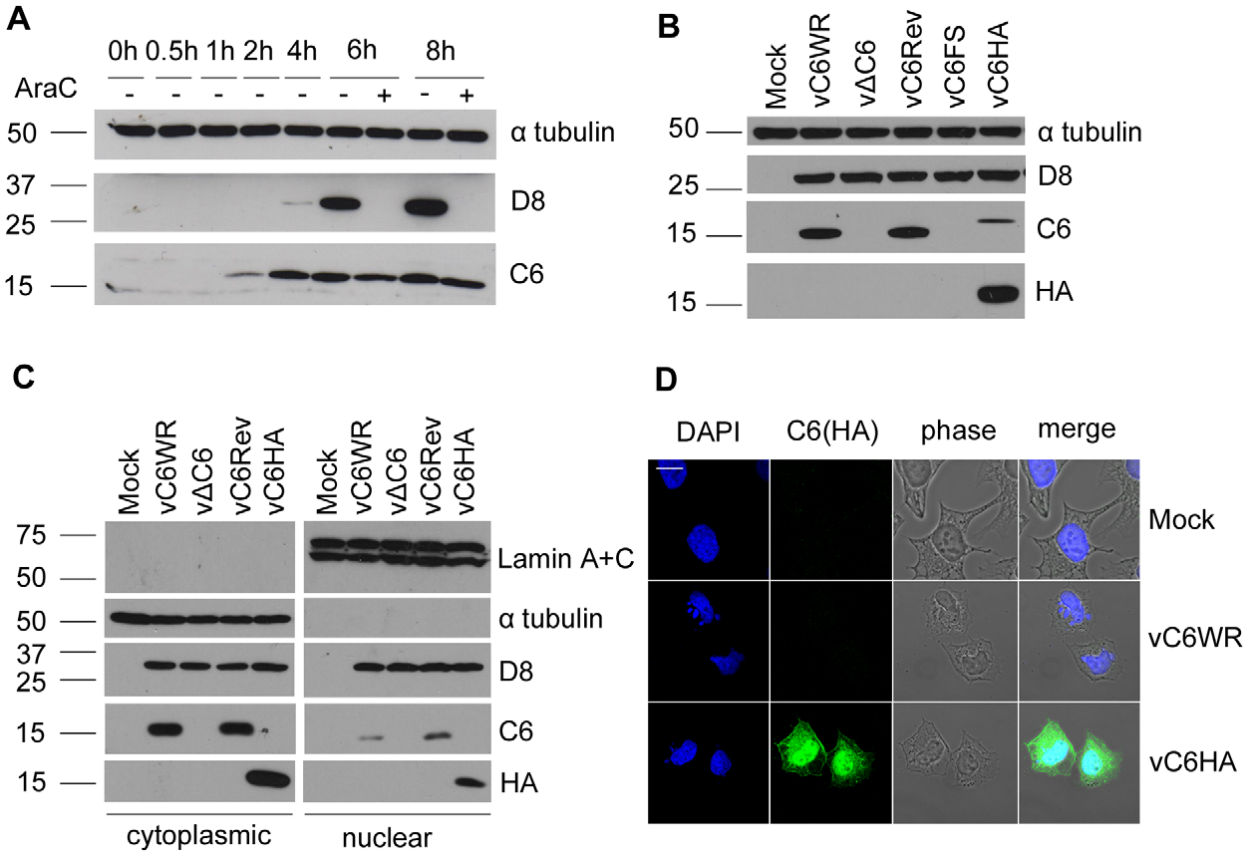
C6 is expressed early during infection and is present in both the cytoplasm and nucleus. (**A**) BSC-1 cells were infected with VACV (5 p.f.u. per cell) in the presence or absence of cytosine arabinoside (AraC) and harvested at the indicated times post infection. Protein extracts were separated by SDS-PAGE and analysed by immunoblotting with the antibodies indicated. Molecular mass markers are indicated on the right (kDa). (**B**) Purified recombinant VACVs were used to infect BSC-1 cells overnight with 5 p.f.u. per cell. Protein extracts were separated by SDS-PAGE and analysed by immunoblotting with the antibodies indicated. (**C**) HeLa cells were infected for 16 h with 5 p.f.u. per cell. Cells were harvested and fractionated into cytoplasmic and nuclear fractions. Proteins from cell extracts were separated by SDS-PAGE and analysed by immunoblotting with the indicated antibodies. Three-fold more of the total nuclear fraction was loaded compared to the total cytoplasmic fraction. (**D**) HeLa cells were infected with the indicated viruses for 5 h with 5 p.f.u. per cell. Cells were then fixed and stained with a mouse anti-HA antibody. The localisation of C6 (green), nuclear and viral DNA stained with DAPI (blue), phase-contrast and merged images are shown.

The expression of C6 protein during infection was investigated by infecting BSC-1 cells with VACV WR in the presence or absence of cytosine arabinoside (AraC), an inhibitor of viral DNA replication and hence intermediate and late protein expression. Using a polyclonal antiserum raised against C6 protein expressed in *Escherichia coli*, a 17-kDa C6 protein was detected starting from 2 h post infection, with continued expression at all time points thereafter (Figure 6A). Also, C6 was detected in the presence of AraC confirming its expression prior to DNA replication and hence as an early protein during infection, consistent with previous data for *C6L* mRNA expression [54]. In contrast, the expression of the late protein D8 [55] was blocked by the presence of AraC.

To characterize the contribution of the C6 protein to VACV replication, spread and virulence, recombinant viruses that did or did not express the C6 protein were generated. These viruses included a *C6L* deletion virus (vΔC6) lacking the C6 ORF, a plaque purified wild type virus (vC6WR) that was isolated from the same intermediate virus as the deletion mutant during transient dominant selection (see methods), a revertant virus where the *C6L* ORF was re-inserted into the deletion virus at its natural locus (vC6Rev), and an additional recombinant virus (vC6FS) where the C6 translational initiation codon was disrupted by the insertion of an adenine nucleotide. As expected, this virus, as well as vΔC6, did not express C6 protein whereas vC6WR and vC6Rev both did (Figure 6B). For localisation and interaction studies a virus expressing HA-tagged C6 was constructed (vC6HA). Reduced levels of C6 were detected from this recombinant virus using the anti-C6 serum, perhaps due to the lower expression of this protein compared to wild-type C6 or poorer detection by the antiserum. Nevertheless this protein was detected using an antibody against the HA epitope (Figure 6B), and shown to be functional in that it was capable of inhibiting IFN-β promoter induction when expressed from a plasmid (data not shown).

The intracellular localisation of C6 was investigated by biochemical fractionation of cells into cytoplasmic and nuclear fractions, followed by immunoblotting using the anti-C6 serum (Figure 6C). The integrity of nuclear and cytoplasmic fractions was confirmed by blotting for lamin A and C, and for tubulin, respectively. The expression of C6 was detected in both nuclear and cytoplasmic fractions of cells infected with the wild-type virus expressing C6 (vC6WR), the revertant virus (vC6Rev) or the virus expressing HA-tagged C6 (vC6HA) (Figure 6C). The anti-C6 serum was not suitable for the detection of wild-type C6 by immunofluorescence. However, both nuclear and cytoplasmic localisation of C6 was also observed by confocal microscopy when cells infected with vC6HA were stained with an antibody against the HA tag (Figure 6D).

### C6 is not required for replication in cell culture

The isolation of the deletion mutant and vC6FS virus indicated that C6 is not essential for VACV replication. To determine whether loss of C6 had an effect on virus replication kinetics or spread, the plaque size and virus growth in cell culture were analysed. The size of the plaques formed 72 h post infection with the various recombinant viruses was measured in three different cell lines: African green monkey BSC-1 cells, rabbit RK-13 cells and human TK-143 cells. The absence of C6 had no effect on the mean plaque size in any of the cell types studied (Figure S3A). To assess viral replication, BSC-1 cells were infected at a high (10) or low (0.01) multiplicity of infection (m. o. i.) with the set of recombinant viruses, and virus in the intracellular and extracellular fractions at various time points post infection was titrated by plaque assay (Figure S3B–E). No significant difference was observed between the titres of either the extracellular or intracellular forms of the recombinant viruses.

### C6 is a VACV virulence factor

**Figure 7:**
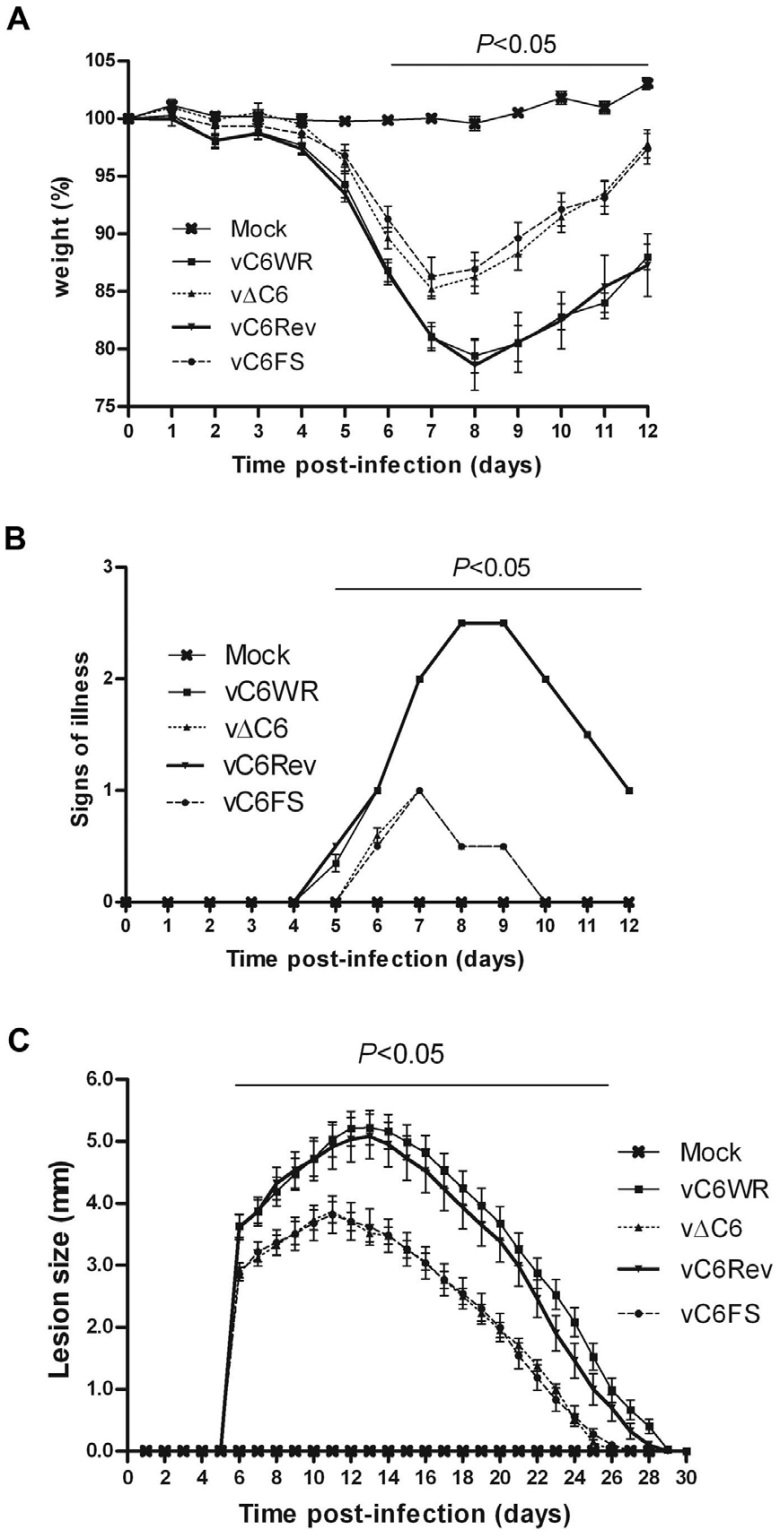
VACV lacking C6 is attenuated *in vivo*. (**A, B**) Virulence in a murine intranasal model of VACV infection. Female BALB/c mice (n = 10) were infected with 5×10^3^ p.f.u. of the indicated viruses and their weights (**A**) and signs of illness (**B**) were monitored daily. Weight data (**B**) are expressed as the percentage ± SEM of the mean weight of the same group of animals on day 0. Signs of illness data (**B**) are expressed as the mean score ± SEM. The horizontal bar indicates the days on which the weight loss or signs of illness induced by vΔC6 and vC6FS were statistically different (*P*<0.05) from both vC6WR and vC6Rev. (**C**) Virulence in murine intradermal model of VACV infection. Female C57BL/6 mice (n = 10) were infected with 10^4^ p.f.u. of the indicated viruses in both ears and the resulting lesions were measured daily. Data are expressed as the mean lesion size ± SEM. The horizontal bar indicates the days on which the lesion size caused by vΔC6 and vC6FS were statistically different (*P*<0.05) from both vC6WR and vC6Rev.

The contribution of C6 to VACV virulence was tested in two murine models of infection. In the intranasal (i.n.) model, groups of 10 BALB/c mice were infected with the recombinant viruses at 5×10^3^ plaque forming units (p.f.u.) per animal and weight loss and signs of illness were recorded and compared. A significant difference in weight loss was observed between the viruses that did not express C6 (vΔC6 and vC6FS) and those that did express C6 (vC6WR and vC6Rev) between days 6 and 12 post infection (Figure 7A), with the mice infected with the C6 deletion viruses losing less weight overall and gaining weight more quickly during recovery. The viruses lacking C6 also caused significantly fewer signs of illness in infected mice between day 5 and day 12 after infection (Figure 7B).

In addition, the recombinant viruses were used to infect groups of 10 BL/6 mice intradermally with 10^4^ p.f.u. virus per ear, in both ears, and the sizes of the resulting lesions were measured and compared. The lesions induced by vΔC6 and vC6FS were significantly smaller than those induced by vC6WR and vC6Rev between 6 and 26 days post infection (Figure 7C). In addition the lesions induced by the viruses lacking C6 began to heal sooner (11 days post infection) than those induced by the viruses that did express C6 (14 days post infection) (Figure 7C). Thus, these data show that a virus lacking C6 is attenuated *in vivo* and indicate C6 is a virulence factor in two different models of infection.

## Discussion

Here the VACV protein C6 is described as a novel modulator of the innate immune system. Data presented show that C6 inhibits IFN-β expression by preventing the activation of the transcription factors IRF3 and IRF7, while not affecting NF-κB activation. C6 acts at the level of the kinases TBK1 and IKKε, and is able to associate with the kinase-associated scaffold proteins NAP1, TANK and SINTBAD. The immunomodulatory function of C6 is likely to be important during infection, as a deletion virus lacking C6 is attenuated in mouse models *in vivo*.

C6 was identified as an inhibitor of the initiation of the IFN-β response in a screen of poorly characterised VACV proteins. That C6 might be an immunomodulator had been suggested by the previous observations that it belongs to a family of poxvirus proteins [37] whose members (A46, A52, B14, N1 and K7) were shown subsequently to belong to the Bcl-2 protein family and to have immunomodulatory activity (reviewed in [38]). While the family members share structural similarity, their degree of amino acid similarity is low indicating they diverged long ago and although they share an ability to manipulate innate immune signalling pathways, they differ in their targets and mechanisms of action. While A46 acts at the interface between TLRs and their adaptors [56], A52 targets the more downstream signalling factors TRAF6 and IL-1 receptor associated kinase 2 (IRAK2) [57]. B14, K7 and now C6 all appear to target the kinase complexes located at the point of convergence between several different PRR signalling pathways. However, each of the viral proteins targets distinct components of these complexes. B14 binds IKKβ and thereby inhibits IκB phosphorylation and NF-κB activation [47]. In contrast, K7 inhibits IRF3 phosphorylation by binding to the helicase DDX3, which is part of the complexes containing TBK1 and IKKε [58].

In this paper C6 is shown to inhibit the activation of IRF3 and IRF7 in a different way to K7, namely by interacting with TANK, NAP1 and SINTBAD. These three proteins act as scaffold proteins that associate constitutively with TBK1 and IKKε [17], [59], [60]. They are essential for the innate immune response to several different viruses and PAMPs, and in particular for the activation of IRFs, but not NF-κB, upon stimulation with Sendai virus or poly(I∶C) [16]–[18]. The observation that the scaffold proteins are targeted by a viral immunomodulator provides additional evidence for the importance of these proteins in the antiviral response. The precise function of TANK, NAP1 and SINTBAD in the process of TBK1 and IKKε activation has not been defined fully, but there is evidence that the adaptor proteins link the kinases to the upstream signalling pathways, possibly by interaction with TRAF3, which is a component of TLR and RLR signalling pathways [16]. The recruitment of the kinase complexes to TRAF3 would then require the scaffold proteins, and lead to the phosphorylation and activation of TBK1 and IKKε, ultimately leading to the phosphorylation of IRF3 and IRF7. However, how exactly complex formation is linked to the activation of the kinases, and which functions of the adaptors may be redundant or unique, has yet to be elucidated.

NAP1, TANK and SINTBAD are related in domain structure, possessing N-terminal coiled-coil regions that are important for homodimerization, and a central TBD, which mediates the interaction with TBK1 and IKKε [17]. While C6 interacted with all three scaffold proteins, it did not affect their interaction with either TBK1 or IKKε. In contrast, truncated proteins containing only the TBD of either NAP1, TANK or SINTBAD inhibited all the scaffold-kinase interactions in this assay, as described previously [17]. Thus the exact mechanism whereby C6 disrupts IRF activation remains to be determined. It is possible that C6 prevents the association of the scaffold-kinase complexes with TRAF3, or else prevents the activation of TBK1 and IKKε once the complexes are formed. Also, it is possible that the interaction between C6 and the scaffold proteins is indirect and is mediated by additional proteins that may be part of the kinase signalling complexes. Further analysis of the effect of C6 on these protein complexes may shed some light on the mechanism by which TBK1 and IKKε are activated - and inhibited - during viral infection.

VACV is not the only virus that inhibits the TRAF3-scaffold-kinase axis. Recently it was shown that the M protein of severe acute respiratory syndrome (SARS) coronavirus targets a complex containing TRAF3, and prevents the association of TRAF3 with TBK1, IKKε or TANK [61]. Like C6, the M protein inhibits the induction of the IFN-β promoter by inhibiting IRF3 activation [61]. However to our knowledge C6 is the first viral protein shown to associate with all three scaffold proteins.

VACV expresses several proteins that inhibit IRF activation, including the related Bcl-2-like protein K7, which also targets a TBK1-containing protein complex [58]. However, despite this, the effects of K7 and C6 are evidently not duplicative, because when K7 is still expressed, loss of C6 caused a marked virus attenuation in two models of infection. Similarly, there are several VACV proteins that inhibit NF-κB activation, for instance A52, A46, N1, B14. Yet deletion of any single member of this group causes virus attenuation suggesting non-redundant functions. Possible explanations for this non-redundancy might be cross talk between different pathways, so the outcome of blocking a pathway is influenced by the point at which a virus inhibitor functions to block the pathway. Alternatively, the virus proteins might have multiple functions as has been demonstrated for VACV protein N1. The need for the virus to express so many different non-redundant viral inhibitors of host signalling cascades may be due to the host innate immune system being able to partially compensate for the inhibition of an individual signalling component by using parallel pathways all ultimately leading to the induction of type I IFNs and pro-inflammatory cytokines. Furthermore, the importance of one particular signalling pathway or component may vary depending on the cell type infected or stage of infection, thus requiring the inhibition of several, seemingly redundant signalling proteins. Finally, it is plausible that the inhibition exerted by a single viral protein in not complete, particularly at early stages of infection, thus requiring the expression of several different factors targeting components of the same pathway to have an additive effect.

The characterization of poxvirus proteins that inhibit the innate immune system is of interest, since elucidating the mechanism of viral inhibition often reveals new insights into how innate immunity operates. Furthermore, VACV strains are in development as vaccine delivery systems against smallpox and other pathogens (reviewed in [62]), and as vectors for the targeting of tumours and for gene therapy (reviewed in [63]). It is shown here that deletion of a single ORF can attenuate the virus, thus possibly making it safer for clinical use. Also, the deletion of ORFs that encode inhibitors of the innate immune system would be predicted to make the virus more immunogenic, and thus make a more effective vaccine. In this context, it is interesting to note that C6 is conserved in most OPVs and that the MPXV orthologue of C6 is functionally equivalent to the C6 protein from VACV strains WR.

## Materials and Methods

### Plasmids

For the screen of VACV candidate immunomodulators, 49 ORFs were selected from the VACV WR strain genome and amplified by PCR from VACV WR genomic DNA isolated by phenol-chloroform extraction from purified viral cores. Candidate ORFs including *C6L* and *B14R* were cloned into the expression vector pCMV-HA (Clontech). C6 was also subcloned into pLENTI-Dest-V5 (Invitrogen) for immunoflourescence experiments. The ORF encoding MPXV C6 was amplified by PCR from DNA extracted from MPXV-infected HeLa cells (a kind gift from K. Rubins, Whitehead Institute) and cloned into pCMV-HA (Clontech). IFN-β-promoter luciferase reporter was a gift from T. Taniguchi (University of Tokyo, Japan) and NF-κB luciferase was from R. Hofmeister (University of Regensburg, Germany). ISRE-Luciferase and pFR-Luciferase were purchased from Promega. GL3-Renilla vector was made by replacing the firefly luciferase ORF from pGL3-control (Promega) with the renilla luciferase ORF from pRL-TK (Promega). FLAG- and luciferase fusions with signalling proteins for the LUMIER assay were described in [17]. IKKα was from Tularik Inc. Vectors expressing IRF3-Gal4, IRF7-Gal4, TBK1, IKKε and TRIF were a kind gift from K.A. Fitzgerald (University of Massachusetts Medical School, USA), MAVS was from T.J. Chen (University of Texas Southwestern Medical Centre, USA), MyD88 was from M Muzio (Milan, Italy), TLR3 was from D. T. Golenbock (University of Massachusetts Medical School, USA), and IRF3-5D and IRF7-4D were from J. Hiscott (McGill University, Montreal, Canada).

For construction of the C6 deletion virus 250-bp flanking regions of the *C6L* gene were amplified by PCR from VACV WR genomic DNA, ligated together and inserted into a plasmid containing the *Escherichia coli guanylphosphoribosyl transferase* (*Ecogpt*) gene fused in-frame with the *enhanced green fluorescent protein* (*EGFP*) gene (Z11ΔC6). For construction of Z11C6rev, Z11C6FS and Z11C6HA, *C6L*, *C6L* with an additional adenine nucleotide in the start codon or *C6L* with a C-terminal HA tag respectively, plus *C6L* flanking regions were amplified from VACV WR genomic DNA and inserted into the Z11 plasmid.

### Antibodies and reagents

C6 polyclonal antiserum was raised against C6 protein purified from *Escherichia coli* and injected into rabbits (Eurogentec). Other antibodies were from the following sources: IRF3 (IBL), V5 (Cell Signaling), p65 (Santa Cruz), IgG from rabbit serum (Sigma), TBK1 (Cell Signaling), IKKε (Abcam), TANK (Abcam), FLAG (Sigma), Lamins A+C (Abcam), tubulin (Upstate Biotech). The mouse monoclonal antibody AB1.1 against D8 has been described previously [64]. Poly(I∶C) and poly(dA-dT) were from Sigma, TNF, IL-1, CLO75 and R848 were from Invivogen.

### Cells

HEK293 cells were grown in Dulbecco's Modified Eagle's Medium (DMEM, GIBCO) supplemented with 10% fetal bovine serum (FBS, Biosera) and 10 µg/ml ciprofloxacin (Sigma). BSC-1 cells were maintained in DMEM supplemented with 10% FBS and penicillin/streptomycin (P/S) (50 µg/ml). RK-13 and TK^-^143 cells were maintained in minimum essential medium (MEM) supplemented with 10% FBS and P/S (50 µg/ml). NIH 3T3 cells were maintained in DMEM supplemented with 10% newborn bovine serum (NBS) and P/S (50 µg/ml). HeLa cells were maintained in MEM supplemented with 10% FBS, 1∶100 non-essential amino acids (NEAA) (Sigma) and P/S (50 µg/ml).

### Construction of recombinant viruses

C6 recombinant viruses were constructed using the transient dominant selection method [65]. For construction of vΔC6, RK-13 cells were infected with VACV strain WR at 0.01 p.f.u. per cell and then transfected with the Z11ΔC6 plasmid using polyethylenimine (PEI) (1 mg/ml) according to the manufacturer's instructions. Progeny virus was harvested after 48 h and used to infect RK-13 cells in the presence of mycophenolic acid (MPA, 25 µg/ml), hypoxanthine (HX, 15 µg/ml) and xanthine (X, 250 µg/ml). EGFP-positive plaques were selected and purified by three rounds of infection using RK-13 cells in the presence of MPA, HX and X as above. Intermediate virus was resolved in BSC-1 cells by three rounds of infection in the absence of MPA, HX and X. The genotype of resolved viruses was analysed by PCR following proteinase K-treatment of infected RK-13 cells. Revertant viruses were constructed in a similar manner by transfection of plasmid Z11C6rev (for vC6 rev), Z11C6FS (for vC6FS) or Z11C6HA (for vC6HA) into vΔC6 infected cells.

### Reporter gene assays

Luciferase reporter gene assays were performed in HEK293 cells seeded in 96-well plates and transfected with 0.8 µl Genejuice (Merck) per well. Firefly reporter plasmid (60 ng), 20 ng GL3-Renilla control plasmid and 150 ng expression vector or empty vector control were used per well. For the IRF3 and IRF7 reporter gene assays, 60 ng pFR-Luciferase and 20 ng pGL3-Renilla were transfected together with 4 ng of IRF3-Gal4 or IRF7-Gal4, and 150 ng expression vector or control plasmid. For luciferase assays in NIH3T3 cells, cells were seeded in 96-well plates and transfected with 60 ng IFN-β-luciferase, 10 ng pRL-TK, and 250 ng expression vector or empty vector control. Cells were lysed in Passive Lysis Buffer (Promega), and firefly luciferase activity was normalized to renilla luciferase activity. Experiments were performed in triplicate and repeated at least 3 times.

### Real-time PCR

RNA from HEK293 cells grown in 12-well plates was extracted using the RNeasy kit (QIAGEN), and converted to cDNA using the Quantitect RT kit (QIAGEN). IFN-β mRNA was quantified by real-time PCR with the TaqMan gene expression assay Hs00277188_s1 and a β-actin endogenous control VIC-MGB probe (6-carboxyrhodamine–minor groove binder; Applied Biosystems). Experiments were performed in triplicate.

### ELISA

Cell culture supernatants from HEK293 cells grown in 96-well plates were assayed for CCL5 protein using Duoset reagents (R&D Biosystems).

### Confocal microscopy

HEK293 cells were grown on glass coverslips and fixed with 4% paraformaldehyde in PBS. Cells were permeabilized in 0.5% Triton in PBS, pre-incubated for 1 h in blocking buffer (5% BSA, 0.05% Tween-20 in PBS), stained for 3 h with primary antibody (1∶300 in blocking buffer) and for 1 h with Alexa488 or Alexa647-labelled secondary antibodies (1∶500, Invitrogen). Coverslips were mounted in MOWIOL 4-88 (Calbiochem) containing DAPI (4,6-diamidino-2-phenylindole; 1 µg/ml). Images were taken on an Olympus FV1000 scanning confocal microscope.

### Co-immunoprecipitation and LUMIER assay

For co-immunoprecipitation, HEK293T cells were grown in 10-cm dishes and co-transfected with vectors expressing FLAG-tagged proteins and a vector expressing TAP-tagged (consisting of FLAG and Streptavidin epitopes) C6, using Fugene-6 (Roche), or transfected with the vectors expressing FLAG-tagged proteins alone and then infected 48 h later with vC6HA (2 p.f.u. per cell) for 16 h. Cells were lysed in lysis buffer (0.1 (v/v) % Triton X-100, 150 mM NaCl, 10% glycerol, 10 mM CaCl_2_, 20 mM Tris-HCl pH 7.4 and protease inhibitors), pre-cleared by centrifugation and incubated with 30 µl of anti-FLAG M2 agarose beads (Sigma), or strepavidin agarose beads (Thermofisher) for 3 h. Immunoprecipitates were washed 3 times in lysis buffer, and eluted from the beads by boiling in sample buffer containing SDS. Proteins were resolved by SDS-polyacrylamide gel electrophoresis (PAGE) and detected by immunoblotting.

For the LUMIER assay, HEK293 cells were grown in 6-well plates and transfected with 0.5 µg FLAG-tagged plasmid, 0.5 µg luciferase-tagged plasmid and 3 µg C6 expression vector, TBD expression vector or empty vector control using GeneJuice (Merck). Cells were harvested 24 h later in Passive Lysis Buffer (Promega), and subjected to immunoprecipitation using 0.3 µl FLAG antibody pre-coupled to Protein A sepharose beads. Immunoprecipitates were washed 5 times in lysis buffer and eluted using 100 µM FLAG peptide (Sigma) in PBS, and renilla luciferase activity was measured.

### Cellular fractionation

HeLa cells were infected with recombinant VACVs at 5 p.f.u. per cell for 16 h. The cells were washed twice in ice-cold LS buffer (20 mM Hepes pH 7.8, 0.5 mM DTT, 0.5 mM MgCl_2_ in water) and allowed to swell on ice for 20 min. The cells were gently scraped and disrupted by Dounce homogenisation on ice. The lysates were centrifuged at 600× *g* for 2 min at 4°C to pellet the nuclei. The supernatant (cytoplasmic fraction) was removed. The nuclei were washed five times in PBS, placed in nuclei resuspension buffer (50 mM Tris-HCl pH 8, 0.5 mM MgCl_2_, 20 mM iodoactetamide supplemented with protease inhibitor (Roche)) and sonicated. Proteins were resolved by SDS-PAGE and detected by immunoblotting.

### *In vitro* growth curves

For the single-step growth curves BSC-1 cells were infected with 10 p.f.u. per cell. At 0 h, 12 h and 24 h post infection the medium was removed and the cells were collected by centrifugation at 500× *g* for 10 min. The supernatant was removed and extracellular virus titres were determined by plaque assay on BSC-1 cells. For intracellular virus, cells were scraped, collected by centrifugation and subjected to three rounds of freeze-thawing before determining viral titre by plaque assay. For the multi-step growth curve BSC-1 cells were infected with 0.01 p.f.u. per cell and intracellular and extracellular virus was harvested at 0, 12, 48 and 72 h post infection as described above.

### Plaque size assay

RK-13, BSC-1 and TK-143 cell monolayers were infected in duplicate with virus at 50 p.f.u. per well for 72 h to allow formation of well separated plaques. The cells were washed once with PBS and stained for 30 min with crystal violet (5% (v/v) crystal violet solution (Sigma), 25% (v/v) ethanol). Wells were then washed with water and the sizes of six plaques per well were measured using Axiovision 4.6 software and a Zeiss Axiovert 200 M microscope.

### Murine intranasal and intradermal models of infection

Female BALB/c mice (n = 10, 6–8 weeks old) were infected intranasally (i.n.) with 5×10^3^ p.f.u. and monitored as described previously [66], [67]. Female C57BL/6 mice (n = 10, 6–8 weeks old) were inoculated intradermally (i.d.) in the ear pinnae with 10^4^ p.f.u. as described previously [68], [69].

### Statistical analysis

Data were analysed using Unpaired Student's T tests. Statistical significance is expressed as follows: * P<0.05, ** P<0.01, *** P<0.001.

### Accession numbers

VACV WR C6, P17362.1; TBK1, AF191838_1; IKKε, NP_054721; NAP1, AAO05967; TANK, NP_001186064; SINTBAD, NP_055541; IKKα, NP_001269; MyD88, AAC50954; MAVS, Q7Z434.2; IRF3, AAH09395; IRF7, NP_001563; NF-κB p65, CAA80524; IFN-β, NC_000009.11; CCL5, NC_000017.10.

## Supporting Information

Figure S1 **C6 function is conserved in monkeypox virus.** (**A, B**) HEK293 cells were seeded in 96-well plates, transfected with a pFR-firefly luciferase reporter plasmid under the control of the Gal4 promoter, a renilla luciferase transfection control, and an IRF3-Gal4 fusion construct. C6 expression vector (50, 100 or 150 ng, wedges) and empty vector (EV) were co-transfected per well. In (**A**), cells were mock transfected or transfected with 500 ng/ml poly(dA-dT) 8 h after the initial transfection. In (**B**), 50 ng plasmids expressing the indicated signalling protein were included in the initial transfection. Cells were harvested 24 h after the first transfection, firefly luciferase activity was measured and normalized to renilla luciferase activity. *p<0.05, ** p<0.01 or ***p<0.001 compared to EV.
(TIF)

Figure S2 **C6 does not prevent the interaction between scaffold proteins and IKKε.** (**A–C**) HEK293 cells were grown in 6-well plates and transfected with 0.5 µg luciferase-tagged IKKε expression construct, 0.5 µg FLAG-tag expression construct and 3 µg C6 expression vector or empty vector (EV) as indicated. Cells were harvested after 24 h and subjected to immunoprecipitation with anti-FLAG antibody. Immunoprecipitated protein complexes were eluted with FLAG peptide and co-immunoprecipitated luciferase activity was measured. Data is representative of at least three experiments.
(TIF)

Figure S3 **C6 deletion does not affect viral replication or spread in tissue culture.** (**A**) Monolayers of BSC-1, RK-13 and TK-143 cells were infected with the indicated viruses for 72 h. Cells were stained with crystal violet and the plaque size was measured using Axiovision 4.6 software and a Zeiss Axiovert 200 M microscope. Results are expressed as the mean plaque radius ± SD. (**B–E**) For construction of single- (**B, C**) and multi-step (**D, E**) growth curves, BSC-1 cells were infected in duplicate at the p.f.u. per cell indicated for 90 min. Unbound virus was washed off, cells were harvested at the indicated time points, and intracellular (**B, D**) and extracellular (**C, E**) virus components were separated. Virus infectivity was titrated by plaque assay on BSC-1 cells. Data are expressed as the mean titre per sample ± SD.
(TIF)

## References

[1] Sadler AJ, Williams BR (2008). Interferon-inducible antiviral effectors.. Nat Rev Immunol.

[2] Stetson DB, Medzhitov R (2006). Type I interferons in host defense.. Immunity.

[3] Hornung V, Ellegast J, Kim S, Brzozka K, Jung A (2006). 5′-Triphosphate RNA Is the Ligand for RIG-I.. Science.

[4] Kato H, Takeuchi O, Sato S, Yoneyama M, Yamamoto M (2006). Differential roles of MDA5 and RIG-I helicases in the recognition of RNA viruses.. Nature.

[5] Pichlmair A, Schulz O, Tan CP, Naslund TI, Liljestrom P (2006). RIG-I-mediated antiviral responses to single-stranded RNA bearing 5′-phosphates.. Science.

[6] Yoneyama M, Kikuchi M, Natsukawa T, Shinobu N, Imaizumi T (2004). The RNA helicase RIG-I has an essential function in double-stranded RNA-induced innate antiviral responses.. Nat Immunol.

[7] O'Neill LA, Bowie AG Sensing and signaling in antiviral innate immunity.. Curr Biol.

[8] Ablasser A, Bauernfeind F, Hartmann G, Latz E, Fitzgerald KA (2009). RIG-I-dependent sensing of poly(dA∶dT) through the induction of an RNA polymerase III-transcribed RNA intermediate.. Nat Immunol.

[9] Burckstummer T, Baumann C, Bluml S, Dixit E, Durnberger G (2009). An orthogonal proteomic-genomic screen identifies AIM2 as a cytoplasmic DNA sensor for the inflammasome.. Nat Immunol.

[10] Chiu YH, Macmillan JB, Chen ZJ (2009). RNA polymerase III detects cytosolic DNA and induces type I interferons through the RIG-I pathway.. Cell.

[11] Fernandes-Alnemri T, Yu JW, Datta P, Wu J, Alnemri ES (2009). AIM2 activates the inflammasome and cell death in response to cytoplasmic DNA.. Nature.

[12] Roberts TL, Idris A, Dunn JA, Kelly GM, Burnton CM (2009). HIN-200 proteins regulate caspase activation in response to foreign cytoplasmic DNA.. Science.

[13] Takaoka A, Wang Z, Choi MK, Yanai H, Negishi H (2007). DAI (DLM-1/ZBP1) is a cytosolic DNA sensor and an activator of innate immune response.. Nature.

[14] Unterholzner L, Keating SE, Baran M, Horan KA, Jensen SB (2010). IFI16 is an innate immune sensor for intracellular DNA.. Nat Immunol.

[15] Hornung V, Ablasser A, Charrel-Dennis M, Bauernfeind F, Horvath G (2009). AIM2 recognizes cytosolic dsDNA and forms a caspase-1-activating inflammasome with ASC.. Nature.

[16] Guo B, Cheng G (2007). Modulation of the interferon antiviral response by the TBK1/IKKi adaptor protein TANK.. J Biol Chem.

[17] Ryzhakov G, Randow F (2007). SINTBAD, a novel component of innate antiviral immunity, shares a TBK1-binding domain with NAP1 and TANK.. EMBO J.

[18] Sasai M, Shingai M, Funami K, Yoneyama M, Fujita T (2006). NAK-associated protein 1 participates in both the TLR3 and the cytoplasmic pathways in type I IFN induction.. J Immunol.

[19] Kawai T, Takahashi K, Sato S, Coban C, Kumar H (2005). IPS-1, an adaptor triggering RIG-I- and Mda5-mediated type I interferon induction.. Nat Immunol.

[20] Meylan E, Curran J, Hofmann K, Moradpour D, Binder M (2005). Cardif is an adaptor protein in the RIG-I antiviral pathway and is targeted by hepatitis C virus.. Nature.

[21] Seth RB, Sun L, Ea C-K, Chen ZJ (2005). Identification and Characterization of MAVS, a Mitochondrial Antiviral Signaling Protein that Activates NF-κB and IRF3.. Cell.

[22] Ishikawa H, Barber GN (2008). STING is an endoplasmic reticulum adaptor that facilitates innate immune signalling.. Nature.

[23] Ishikawa H, Ma Z, Barber GN (2009). STING regulates intracellular DNA-mediated, type I interferon-dependent innate immunity.. Nature.

[24] Oshiumi H, Sasai M, Shida K, Fujita T, Matsumoto M (2003). TIR-containing adapter molecule (TICAM)-2, a bridging adapter recruiting to toll-like receptor 4 TICAM-1 that induces interferon-beta.. J Biol Chem.

[25] Yamamoto M, Sato S, Hemmi H, Hoshino K, Kaisho T (2003). Role of adaptor TRIF in the MyD88-independent toll-like receptor signaling pathway.. Science.

[26] Bowie AG (2010). TRAF3: uncovering the real but restricted role in human.. Immunity.

[27] Au WC, Moore PA, Lowther W, Juang YT, Pitha PM (1995). Identification of a member of the interferon regulatory factor family that binds to the interferon-stimulated response element and activates expression of interferon-induced genes.. Proc Natl Acad Sci U S A.

[28] Grandvaux N, Servant MJ, tenOever B, Sen GC, Balachandran S (2002). Transcriptional profiling of interferon regulatory factor 3 target genes: direct involvement in the regulation of interferon-stimulated genes.. J Virol.

[29] Honda K, Yanai H, Negishi H, Asagiri M, Sato M (2005). IRF-7 is the master regulator of type-I interferon-dependent immune responses.. Nature.

[30] Sato M, Suemori H, Hata N, Asagiri M, Ogasawara K (2000). Distinct and essential roles of transcription factors IRF-3 and IRF-7 in response to viruses for IFN-alpha/beta gene induction.. Immunity.

[31] Kawai T, Sato S, Ishii KJ, Coban C, Hemmi H (2004). Interferon-alpha induction through Toll-like receptors involves a direct interaction of IRF7 with MyD88 and TRAF6.. Nat Immunol.

[32] Hoshino K, Sugiyama T, Matsumoto M, Tanaka T, Saito M (2006). IkappaB kinase-alpha is critical for interferon-alpha production induced by Toll-like receptors 7 and 9.. Nature.

[33] Muller U, Steinhoff U, Reis LF, Hemmi S, Pavlovic J (1994). Functional role of type I and type II interferons in antiviral defense.. Science.

[34] Randall RE, Goodbourn S (2008). Interferons and viruses: an interplay between induction, signalling, antiviral responses and virus countermeasures.. J Gen Virol.

[35] Bowie AG, Unterholzner L (2008). Viral evasion and subversion of pattern-recognition receptor signalling.. Nat Rev Immunol.

[36] Perdiguero B, Esteban M (2009). The interferon system and vaccinia virus evasion mechanisms.. J Interferon Cytokine Res.

[37] Smith GL, Chan YC, Howard ST (1991). Nucleotide sequence of 42 kbp of vaccinia virus strain WR from near the right inverted terminal repetition.. J Gen Virol.

[38] Gonzalez JM, Esteban M (2010). A poxvirus Bcl-2-like gene family involved in regulation of host immune response: sequence similarity and evolutionary history.. Virol J.

[39] Oda S, Schroder M, Khan AR (2009). Structural basis for targeting of human RNA helicase DDX3 by poxvirus protein K7.. Structure.

[40] Graham SC, Bahar MW, Cooray S, Chen RA, Whalen DM (2008). Vaccinia virus proteins A52 and B14 Share a Bcl-2-like fold but have evolved to inhibit NF-kappaB rather than apoptosis.. PLoS Pathog.

[41] Aoyagi M, Zhai D, Jin C, Aleshin AE, Stec B (2007). Vaccinia virus N1L protein resembles a B cell lymphoma-2 (Bcl-2) family protein.. Protein Sci.

[42] Cooray S, Bahar MW, Abrescia NG, McVey CE, Bartlett NW (2007). Functional and structural studies of the vaccinia virus virulence factor N1 reveal a Bcl-2-like anti-apoptotic protein.. J Gen Virol.

[43] Kvansakul M, Yang H, Fairlie WD, Czabotar PE, Fischer SF (2008). Vaccinia virus anti-apoptotic F1L is a novel Bcl-2-like domain-swapped dimer that binds a highly selective subset of BH3-containing death ligands.. Cell Death Differ.

[44] Kalverda AP, Thompson GS, Vogel A, Schroder M, Bowie AG (2008). Poxvirus K7 Protein Adopts a Bcl-2 Fold: Biochemical Mapping of Its Interactions with Human DEAD Box RNA Helicase DDX3.. J Mol Biol.

[45] DiPerna G, Stack J, Bowie AG, Boyd A, Kotwal G (2004). Poxvirus protein N1L targets the I-kappaB kinase complex, inhibits signaling to NF-kappaB by the tumor necrosis factor superfamily of receptors, and inhibits NF-kappaB and IRF3 signaling by toll-like receptors.. J Biol Chem.

[46] Gubser C, Hue S, Kellam P, Smith GL (2004). Poxvirus genomes: a phylogenetic analysis.. J Gen Virol.

[47] Chen RA, Ryzhakov G, Cooray S, Randow F, Smith GL (2008). Inhibition of IkappaB kinase by vaccinia virus virulence factor B14.. PLoS Pathog.

[48] Lin R, Heylbroeck C, Pitha PM, Hiscott J (1998). Virus-dependent phosphorylation of the IRF-3 transcription factor regulates nuclear translocation, transactivation potential, and proteasome-mediated degradation.. Mol Cell Biol.

[49] Honda K, Taniguchi T (2006). IRFs: master regulators of signalling by Toll-like receptors and cytosolic pattern-recognition receptors.. Nat Rev Immunol.

[50] Kawai T, Sato S, Ishii KJ, Coban C, Hemmi H (2004). Interferon-alpha induction through Toll-like receptors involves a direct interaction of IRF7 with MyD88 and TRAF6.. Nat Immunol.

[51] Thurston TL, Ryzhakov G, Bloor S, von Muhlinen N, Randow F (2009). The TBK1 adaptor and autophagy receptor NDP52 restricts the proliferation of ubiquitin-coated bacteria.. Nat Immunol.

[52] Clark K, Plater L, Peggie M, Cohen P (2009). Use of the pharmacological inhibitor BX795 to study the regulation and physiological roles of TBK1 and IkappaB kinase epsilon: a distinct upstream kinase mediates Ser-172 phosphorylation and activation.. J Biol Chem.

[53] Barrios-Rodiles M, Brown KR, Ozdamar B, Bose R, Liu Z (2005). High-throughput mapping of a dynamic signaling network in mammalian cells.. Science.

[54] Assarsson E, Greenbaum JA, Sundstrom M, Schaffer L, Hammond JA (2008). Kinetic analysis of a complete poxvirus transcriptome reveals an immediate-early class of genes.. Proc Natl Acad Sci U S A.

[55] Niles EG, Seto J (1988). Vaccinia virus gene D8 encodes a virion transmembrane protein.. J Virol.

[56] Stack J, Haga IR, Schroder M, Bartlett NW, Maloney G (2005). Vaccinia virus protein A46R targets multiple Toll-like-interleukin-1 receptor adaptors and contributes to virulence.. J Exp Med.

[57] Harte MT, Haga IR, Maloney G, Gray P, Reading PC (2003). The poxvirus protein A52R targets Toll-like receptor signaling complexes to suppress host defense.. J Exp Med.

[58] Schroder M, Baran M, Bowie AG (2008). Viral targeting of DEAD box protein 3 reveals its role in TBK1/IKKepsilon-mediated IRF activation.. EMBO J.

[59] Fujita F, Taniguchi Y, Kato T, Narita Y, Furuya A (2003). Identification of NAP1, a regulatory subunit of IkappaB kinase-related kinases that potentiates NF-kappaB signaling.. Mol Cell Biol.

[60] Pomerantz JL, Baltimore D (1999). NF-kappaB activation by a signaling complex containing TRAF2, TANK and TBK1, a novel IKK-related kinase.. EMBO J.

[61] Siu KL, Kok KH, Ng MH, Poon VK, Yuen KY (2009). Severe acute respiratory syndrome coronavirus M protein inhibits type I interferon production by impeding the formation of TRAF3.TANK.TBK1/IKKepsilon complex.. J Biol Chem.

[62] Jacobs BL, Langland JO, Kibler KV, Denzler KL, White SD (2009). Vaccinia virus vaccines: past, present and future.. Antiviral Res.

[63] Kirn DH, Thorne SH (2009). Targeted and armed oncolytic poxviruses: a novel multi-mechanistic therapeutic class for cancer.. Nat Rev Cancer.

[64] Parkinson JE, Smith GL (1994). Vaccinia virus gene A36R encodes a M(r) 43–50 K protein on the surface of extracellular enveloped virus.. Virology.

[65] Falkner FG, Moss B (1988). Escherichia coli gpt gene provides dominant selection for vaccinia virus open reading frame expression vectors.. J Virol.

[66] Williamson JD, Reith RW, Jeffrey LJ, Arrand JR, Mackett M (1990). Biological characterization of recombinant vaccinia viruses in mice infected by the respiratory route.. J Gen Virol.

[67] Alcami A, Smith GL (1992). A soluble receptor for interleukin-1 beta encoded by vaccinia virus: a novel mechanism of virus modulation of the host response to infection.. Cell.

[68] Tscharke DC, Smith GL (1999). A model for vaccinia virus pathogenesis and immunity based on intradermal injection of mouse ear pinnae.. J Gen Virol.

[69] Tscharke DC, Reading PC, Smith GL (2002). Dermal infection with vaccinia virus reveals roles for virus proteins not seen using other inoculation routes.. J Gen Virol.

