# Supplementary figures and images for: Vaccinia Virus Protein C6 Is a Virulence Factor that Binds TBK-1 Adaptor Proteins and Inhibits Activation of IRF3 and IRF7
Source: PLoS Pathog. 2011 Sep 8;7(9):e1002247. doi: 10.1371/journal.ppat.1002247 (PMC3169548; doi:10.1371/journal.ppat.1002247)

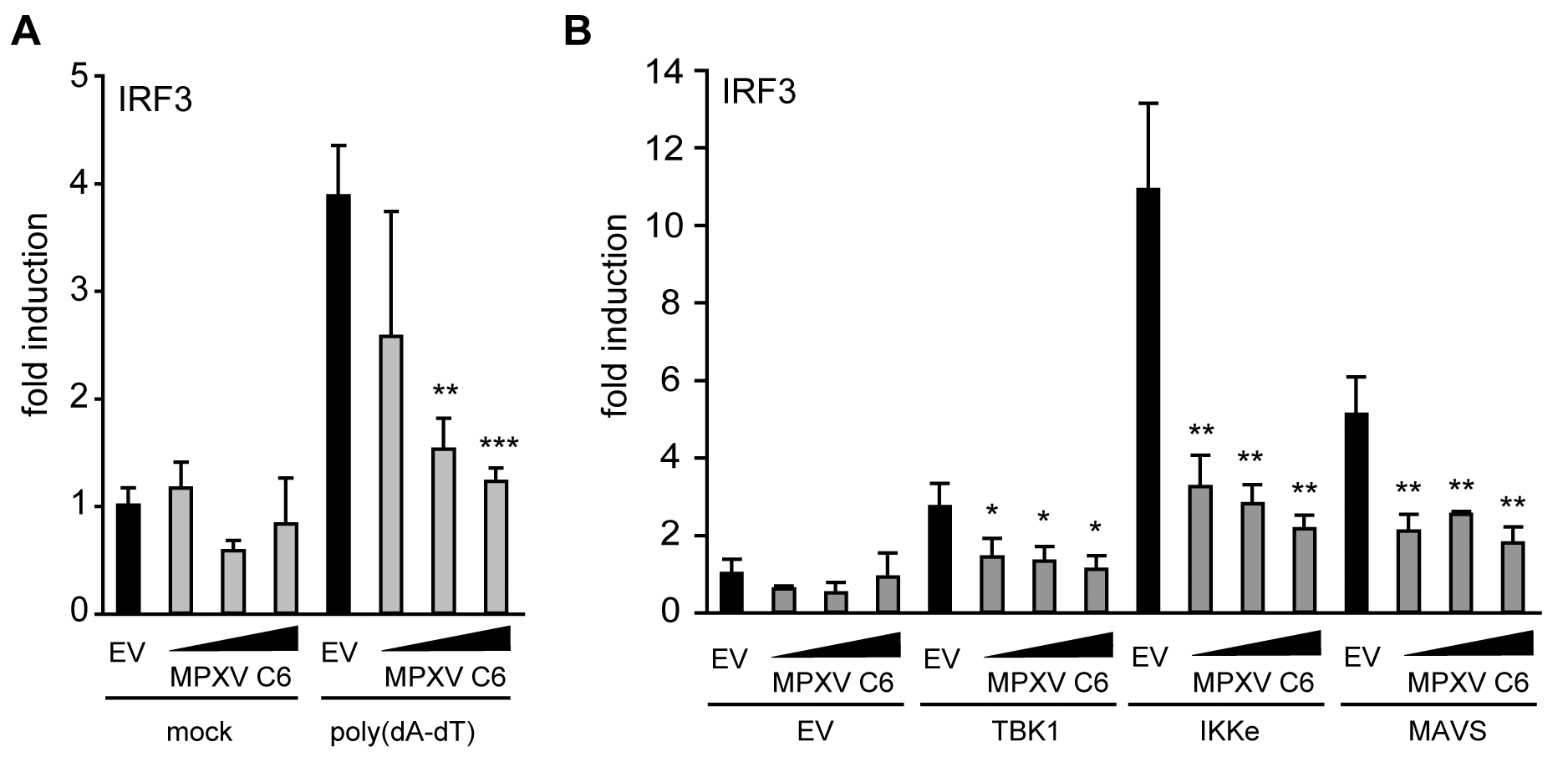

Supplement: Figure S1 — C6 function is conserved in monkeypox virus. (A, B) HEK293 cells were seeded in 96-well plates, transfected with a pFR-firefly luciferase reporter plasmid under the control of the Gal4 promoter, a renilla luciferase transfection control, and an IRF3-Gal4 fusion construct. C6 expression vector (50, 100 or 150 ng, wedges) and empty vector (EV) were co-transfected per well. In (A), cells were mock transfected or transfected with 500 ng/ml poly(dA-dT) 8 h after the initial transfection. In (B), 50 ng plasmids expressing the indicated signalling protein were included in the initial transfection. Cells were harvested 24 h after the first transfection, firefly luciferase activity was measured and normalized to renilla luciferase activity. *p<0.05, ** p<0.01 or ***p<0.001 compared to EV. (TIF) [file ppat.1002247.s001.tif]

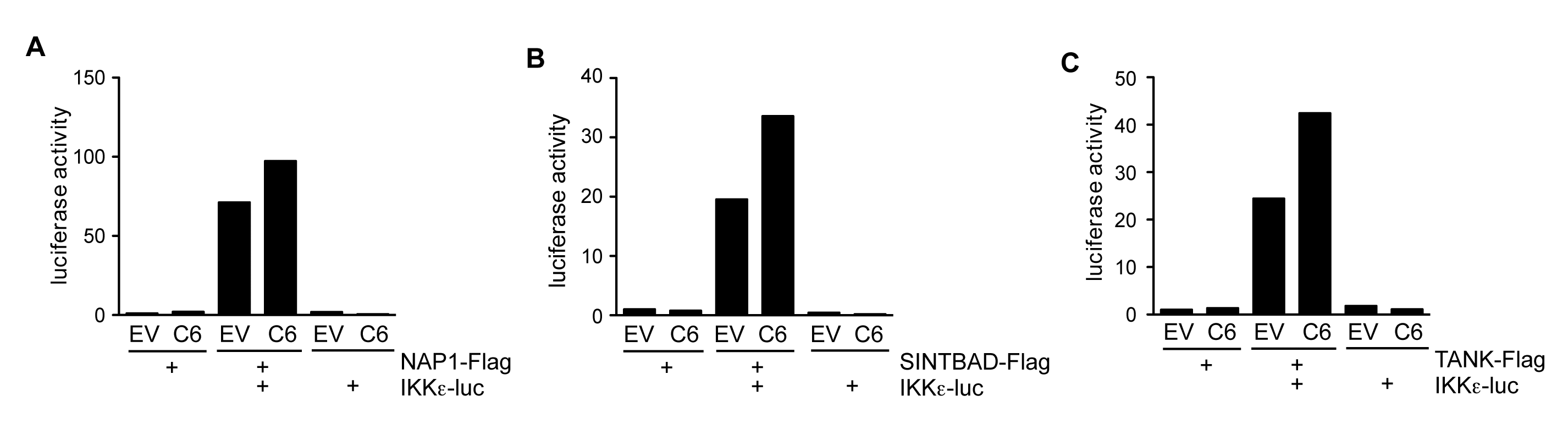

Supplement: Figure S2 — C6 does not prevent the interaction between scaffold proteins and IKKε. (A–C) HEK293 cells were grown in 6-well plates and transfected with 0.5 µg luciferase-tagged IKKε expression construct, 0.5 µg FLAG-tag expression construct and 3 µg C6 expression vector or empty vector (EV) as indicated. Cells were harvested after 24 h and subjected to immunoprecipitation with anti-FLAG antibody. Immunoprecipitated protein complexes were eluted with FLAG peptide and co-immunoprecipitated luciferase activity was measured. Data is representative of at least three experiments. (TIF) [file ppat.1002247.s002.tif]

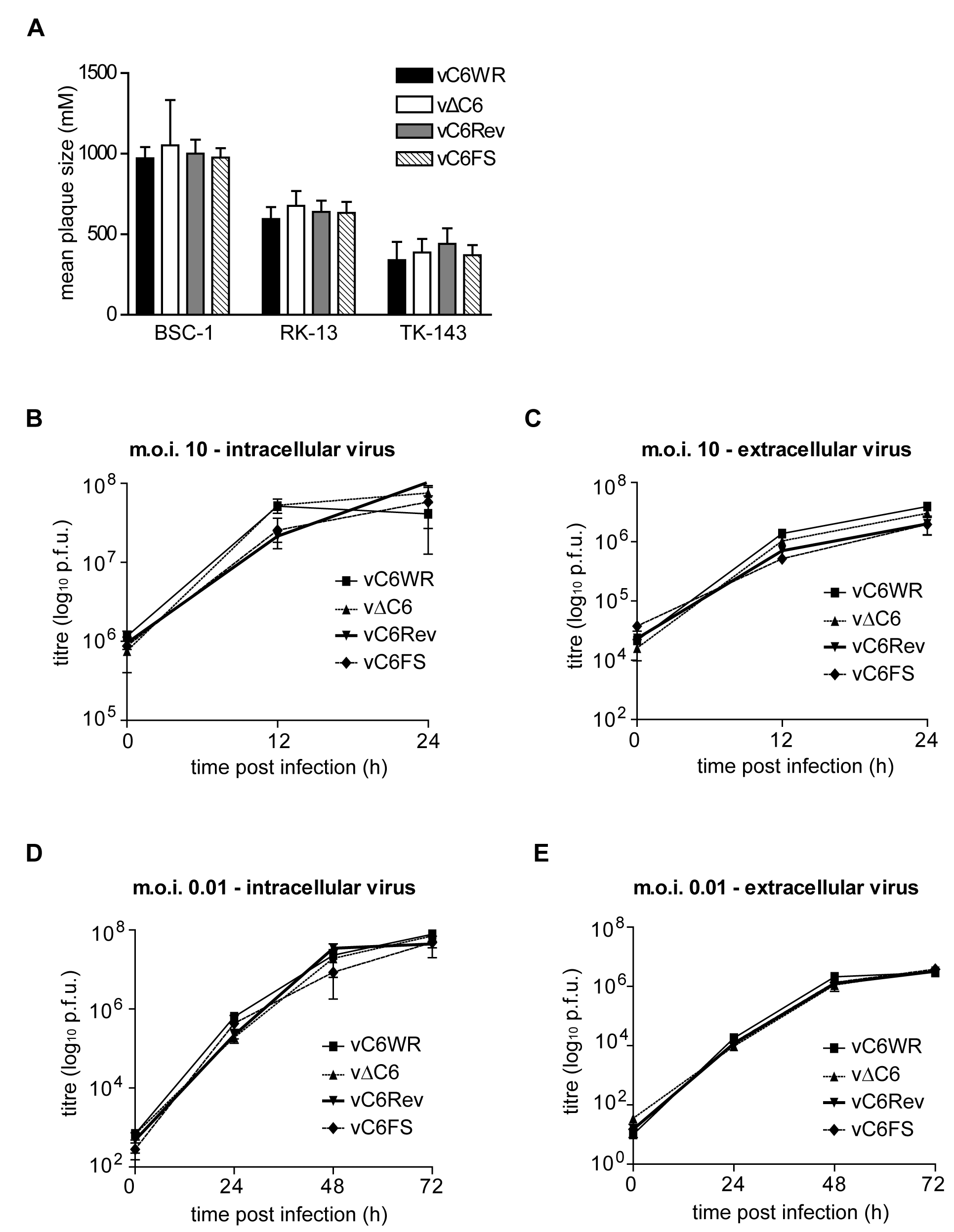

Supplement: Figure S3 — C6 deletion does not affect viral replication or spread in tissue culture. (A) Monolayers of BSC-1, RK-13 and TK-143 cells were infected with the indicated viruses for 72 h. Cells were stained with crystal violet and the plaque size was measured using Axiovision 4.6 software and a Zeiss Axiovert 200 M microscope. Results are expressed as the mean plaque radius ± SD. (B–E) For construction of single- (B, C) and multi-step (D, E) growth curves, BSC-1 cells were infected in duplicate at the p.f.u. per cell indicated for 90 min. Unbound virus was washed off, cells were harvested at the indicated time points, and intracellular (B, D) and extracellular (C, E) virus components were separated. Virus infectivity was titrated by plaque assay on BSC-1 cells. Data are expressed as the mean titre per sample ± SD. (TIF) [file ppat.1002247.s003.tif]
